# The impact of hydrocortisone treatment on neutrophil gelatinase-associated lipocalin release in porcine endotoxemic shock

**DOI:** 10.1186/s40635-017-0117-6

**Published:** 2017-01-19

**Authors:** E. Söderberg, M. Eriksson, A. Larsson, M. Lipcsey

**Affiliations:** 1 Anaesthesiology and Intensive Care, Department of Surgical Sciences, Uppsala University, 751 85 Uppsala, Sweden; 2 Section of Clinical Chemistry, Department of Medical Sciences, Uppsala University, Uppsala, Sweden; 3 Hedenstierna laboratory, Anaesthesiology and Intensive Care, Department of Surgical Sciences, Uppsala University, Uppsala, Sweden

**Keywords:** Hydrocortisone, Sepsis, Endotoxins, Pigs, Neutrophils, NGAL

## Abstract

**Background:**

A key feature of sepsis is systemic inflammatory activation that could be counteracted by steroids. In this experimental model of systemic inflammation, we sought to investigate whether septic neutrophil activation, evaluated by the plasma levels of neutrophil gelatinase-associated protein (NGAL), is modulated by the timing of hydrocortisone treatment.

**Methods:**

Sixteen anesthetized pigs were allocated to one of four equally sized groups. Three of these groups received endotoxin at 2 μg × kg^−1^ × h^−1^ for 6 h so as to induce endotoxemic shock. Hydrocortisone (5 mg × kg^−1^) was administered intravenously before endotoxemic challenge, or at the onset of endotoxemic shock. Endotoxemic pigs not receiving hydrocortisone and non-endotoxemic pigs served as control groups. Physiologic variables, hematology, and biochemistry, including plasma NGAL, were measured repeatedly.

**Results:**

Hydrocortisone treatment prior to endotoxemia attenuated some inflammatory, hematological, circulatory, and metabolic manifestations of shock (i.e., higher white blood cell count, higher mean arterial pressure, lower heart rate and mean pulmonary arterial pressure, higher left ventricular stroke work index, higher base excess). Endotoxemic shock increased plasma NGAL (*p* < 0.001). In pigs given hydrocortisone before the endotoxin infusion, plasma NGAL was lower as compared to those given hydrocortisone at endotoxemic shock (*p* < 0.05). Plasma NGAL levels correlated inversely to neutrophil granulocyte counts (rho = −0.65) but not to urine output (rho = −0.10) at the end of the experiment.

**Conclusions:**

The increase in plasma NGAL is counteracted by hydrocortisone administration prior to endotoxemia; concomitantly, this treatment was associated with less expressed circulatory derangement. Urine NGAL did not differ between the groups, suggesting that the NGAL response was not primarily related to kidney injury.

## Background

Septic shock is associated with significant mortality despite the use of appropriate antibiotics, adequate resuscitation, and modern intensive care [1]. Sepsis and septic shock are complex systemic inflammatory syndromes with potentially deleterious host responses to infection associated with organ dysfunction [2].

White blood cells, in particular the neutrophil granulocytes, are important and active components in this response. Neutrophil granulocyte activation leads to release of granules containing inflammatory meditators, and this process is considered to be a part of anti-microbial defense mechanisms [2, 3].

Among several mediators released by neutrophil granulocytes, neutrophil gelatinase-associated protein (NGAL) in urine has attracted interest as a biomarker of inflammation and acute kidney injury (AKI) [4]. NGAL is a member of the lipocalin family of proteins having capacity to bind bacterial catecholate-type ferric siderophores and act as a potent bacteriostatic agent [5]. NGAL, also known as human neutrophil lipocalin (HNL) or lipocalin 2, was originally identified as a protein released following neutrophil activation [6]. NGAL appears early in granulocyte differentiation as a marker of neutrophil formation [7]. Although NGAL has been in focus for many years as a marker of AKI, recent data suggests that its ability to distinguish between inflammatory activation and AKI is poor [8], and in many instances, plasma NGAL levels are to a greater extent related to the inflammatory response rather than to AKI. Thus, NGAL is a marker of inflammation, specifically, a marker of neutrophil granulocyte activation in plasma.

Steroids, specifically glucocorticoids, may be useful in the treatment of septic shock, and one possible mechanism of action could be attenuation of the inflammatory response.

We hypothesized, based on previous experimental [9–11] and clinical data [12], that the anti-inflammatory activity of steroids is mediated through attenuation of innate immune system activity, specifically the attenuation of neutrophil activation.

To investigate this, we used an experimental model of sepsis [13–15] inducing the systemic inflammatory response and neutrophil granulocyte activation, by administration of endotoxin. The impact of hydrocortisone treatment on the activation of these cells was evaluated by measurement of NGAL in plasma.

Our primary endpoint was to investigate whether NGAL levels differ between endotoxemic pigs treated with hydrocortisone prior to and after the induction of endotoxemia comparing them to endotoxemic pigs without hydrocortisone treatment. The secondary endpoint was to describe the time course of NGAL levels in endotoxemic and non-endotoxemic pigs. We also wished to describe changes in physiology in these animals.

## Methods

### Animals and concession

Sixteen apparently healthy pigs of male gender, 9–11 weeks old, weighing between 23.0 and 30.2 kg (mean ± SD, 27.0 ± 2.1 kg), were included in this study. All pigs had food and water ad libitum until 1 h before preparatory procedures. The following criteria were applied for inclusion: no obvious pre-existing disease, mean pulmonary arterial pressure (MPAP) <2.67 kPa (20 mmHg), and PaO_2_ of >10 kPa (75 mmHg) at baseline, which was 30 min after having accomplished preparatory procedures. The Animals Ethics Committee of the Swedish animal welfare agency, Sweden, approved the experiments (C225/8), and all animals were handled according to the guidelines of the Swedish National Board for Laboratory Animals and the European Convention on Animal Care.

### Anesthesia, fluid administration, and preparatory procedures

All pigs were given an intramuscular injection of 50 mg xylazin as pre-medication before transport from the breeder, and anesthetized as described earlier in detail by us [10–12]. In brief, general anesthesia was induced by injecting 6 mg × kg^−1^ tilétamin-zolazepam intramuscularly. Anesthesia was then maintained with sodium pentobarbital 8 mg × kg^−1^ × h^−1^ mixed with pancuronium bromide 0.26 mg × kg^−1^ × h^−1^ and morphine 0.48 mg × kg^−1^ × h^−1^ dissolved in a 2.5% glucose solution with electrolytes, which was given as a continuous infusion at a rate of 8 mL × kg^−1^ × h^−1^ together with an infusion of sodium chloride, 9 mg × mL^−1^, at a rate of mL × h^−1^, resulting in a total fluid administration of 30 mL × kg^−1^ × h^−1^ during the experiment.

Before securing the airway with a tracheotomy, boluses of 20 mg morphine and 100 mg ketamine were given intravenously. The animals were mechanically ventilated (Servo 900C, Siemens-Elema, Stockholm, Sweden), FiO_2_: 0.3 in medical air was delivered throughout the experiment, and respiratory status was followed with arterial blood samples. The respiratory rate was initially set to 25 × min^−1^. After completion of the preparation procedure, the ventilation was adjusted to yield a PaCO_2_ between 38 and 41 torr (5.0–5.5 kPa). A cervical artery was catheterized for pressure monitoring and blood sampling. A central venous catheter and a Swan-Ganz catheter were inserted through the external jugular vein into the superior caval vein and the pulmonary artery, respectively. Left ventricular stroke work index (LVSWI) and cardiac index (CI) were derived from their conventional formulas. A urinary catheter was introduced into the bladder through a vesicotomy. A heating pad (Operatherm 200W, KanMed, Bromma, Sweden) was set to 38 °C throughout the experiment to decrease heat losses. As soon as the preparation was completed, the pigs were placed in prone position and a 30-min stabilization time passed before baseline values (0 h) were registered and baseline blood samples were collected, after which the endotoxin infusion, or corresponding amount of saline, was started. Measurements of respiratory and circulatory values and blood sampling were made hourly thereafter until the end of the experiment.

### Protocol

The pigs were allocated to one out of four equally sized groups, designed as a modified Latin square, by an unbiased animal laboratory assistant. The pigs received endotoxin, hydrocortisone, or saline in various combinations, in order to unveil potential differences in the endotoxemic response on NGAL, related to the timing of hydrocortisone administration. The chosen steroid dose (5 mg × kg^−1^) is in the same range as previously used, both in clinical and experimental settings [16–18]. Endotoxemic shock was induced by a 6-h continuous infusion of endotoxin at 2.0 μg × kg^−1^ × h^−1^ (*Escherichia coli*: 0111:B4; Sigma Chemical, St. Louis, MO). The endotoxin dose was selected from a previous study of ours [14]. The four groups comprised the following: one group (Etx + Hct; *n* = 4) was given a single intravenous dose of hydrocortisone, at the onset of endotoxemic shock, defined as the moment when the mean pulmonary arterial pressure (MPAP) reached double the baseline value [18]. Doubling of MPAP is a sign of endotoxemia in this model. Another group (Hct + Etx; *n* = 4) received hydrocortisone 30 min before the start of the endotoxin infusion. A third group (Etx + NaCl; *n* = 4) was given the same amount of endotoxin, but the corresponding volume of saline was administered instead of hydrocortisone. The fourth group (NaCl + NaCl; *n* = 4) served as a control group receiving normal saline twice instead of endotoxin or hydrocortisone.

As the endpoint of the experiment was to study NGAL levels during 6 h of endotoxemia, the pigs that did not survive to the end of the experiment were not included in the study. Thus, allocation of new experimental animals into the study continued until the predefined group size of four animals per group was reached.

### NGAL porcine specific assay

NGAL in plasma and urine were analyzed using a Pig NGAL ELISA (Kit 044, BioPorto, Gentofte, Denmark). Samples and calibrators were added to a microtiter plate coated with mouse monoclonal antibodies against pig NGAL. Bound NGAL was then detected with another mouse monoclonal antibody labeled with biotin and a streptavidine-HRP conjugate. The samples were analyzed as singletons in batch mode. The total coefficient of variation for the method in this study was estimated at 5%. Urine NGAL levels were corrected for hourly urine output in each animal.

### Statistics

Data was tested for normality and for baseline differences between groups. Plasma NGAL, heart rate, plasma lactate, and WBC data were log-transformed to achieve normal distribution. Intergroup differences were assessed with repeated measures ANOVA type III for normally distributed data. If group difference was found, a post hoc test with unequal *N* test for groups Etx + NaCl vs. Hct + Etx, groups Etx + NaCl vs. Etx + Hct, and groups Etx + Hct vs. Hct + Etx was performed. Intergroup differences were assessed for non-normally distributed data with Kruskal-Wallis test. Change over time, for non-normally distributed data, was assessed with Friedman’s ANOVA. A Spearman Rank Order test was performed to analyze correlation between data. *p* < 0.05 was considered significant. We report values as means with standard deviation (SD) or medians with interquartile ranges (IQRs) when appropriate. Analyses were performed by using STATISTICA software, version 13 (StatSoft, Tulsa, OK).

## Results

All pigs fulfilled the inclusion criteria. Two pigs died during the experiment. The first pig (Etx + NaCl) died from circulatory collapse after 1 h and the second pig (Etx + Hct) died at the end of the experiment. Both pigs that died before the end of the experiment were replaced at a later date as the endpoint of the experiment was to investigate levels of plasma NGAL during 6 h of endotoxemia.

### NGAL

**Fig. 1:**
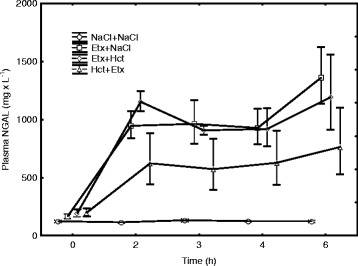
The hourly evolution of plasma NGAL during the experiment in endotoxemic (Etx) and non-endotoxemic pigs (NaCl + NaCl). Plasma NGAL levels were higher during all three endotoxemic groups (1–6 h) than in the NaCl + NaCl (*p* < 0.001) and were higher in the Etx + NaCl and the Etx + Hct group compared in the Hct + Etx during endotoxemia (*p* < 0.05 for both). Mean ± SEM. The curves are depicted with a slight shift to avoid overlap

At baseline, plasma NGAL levels were similar in the four groups (Fig. 1). Plasma NGAL levels increased in all endotoxemic groups, and levels were higher during endotoxemia than in the NaCl + NaCl group (*p* < 0.001). Moreover, levels of plasma NGAL were higher in the Etx + NaCl and the Etx + Hct group compared in the Hct + Etx group during endotoxemia (*p* < 0.05 for both).

Urine NGAL was lower in non-endotoxemic animals compared in each endotoxemic group (*p* < 0.001 for all) but did not differ between the endotoxemic groups during the experiment.

Plasma NGAL levels correlated inversely to neutrophil granulocyte counts (rho = −0.65) and WBC (rho = −0.59) but not to urine output (mls × h^−1^; rho = −0.10) at the end of the experiment.

### Blood cell counts

Pigs in the Hct + Etx group had higher WBC levels than the other groups at baseline (*p* < 0.05), 30 min after administration of hydrocortisone but before endotoxin infusion was started. WBC decreased in each endotoxemic group, but not in non-endotoxemic controls (NaCl + NaCl, Table 1). WBC was lower in each endotoxemic group when compared to that in the NaCl + NaCl group (*p* < 0.001) and also when compared to those in the groups pretreated with hydrocortisone (Etx + NaCl vs. Hct + Etx, *p* < 0.01; Etx + Hct vs. Hct + Etx, *p* < 0.01) during endotoxemia.

**Table 1:**
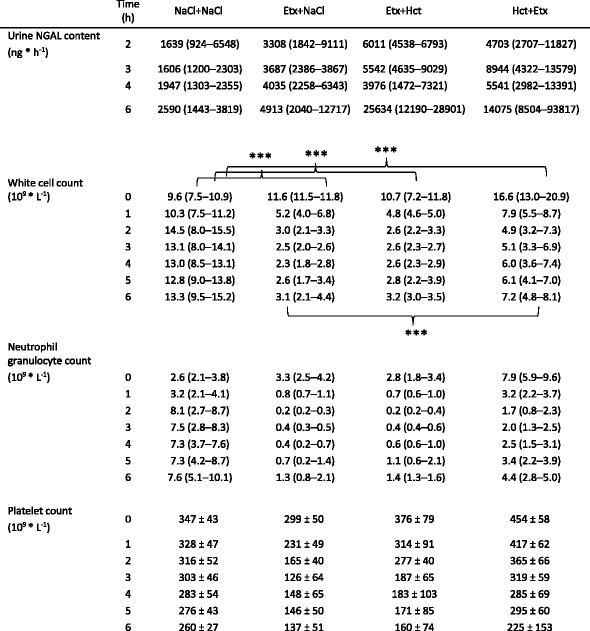
Hematology during 6 h of endotoxemia. Mean ± SD or median (IQR)

***p < 0.001 denotes intergroup differences 1–6 h

Platelets differed between the Etx + NaCl and Hct + Etx groups at baseline, *p* < 0.05. This intergroup difference was also seen during the experiment.

### Circulation

**Fig. 2:**
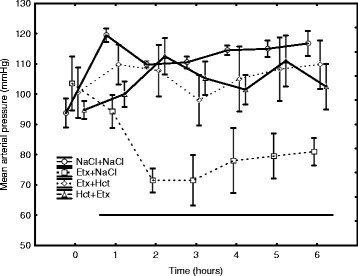
Mean arterial pressure (MAP) hourly during the experiment in endotoxemic (Etx) and non-endotoxemic pigs (NaCl + NaCl). The *dotted line* shows the period when MAP was lower in the Etx + NaCl group compared to each of the other groups (*p* < 0.001 for all). Mean ± SEM. The curves are depicted with a slight shift to avoid overlap

The trajectory of heart rate (HR) was different between the groups during the experiment (Table 2). Post hoc analyses showed that HR was higher in each endotoxemic group vs. NaCl + NaCl controls (*p* < 0.001 for all) and was lower in Hct + Etx vs. both Etx + Hct (*p* < 0.01) and Etx + NaCl (*p* < 0.01) groups. Similarly, mean arterial pressure (MAP) was lower in the Etx + NaCl group than in the other groups (*p* < 0.001; Fig. 2). Other circulatory data are presented in Table 2. Mean pulmonary arterial pressure (MPAP) was higher in each endotoxemic group compared to the NaCl + NaCl group (*p* < 0.01) as it was in the Etx + NaCl group vs. the Etx + Hct and the Hct + Etx groups (*p* < 0.01 for both). Cardiac index (CI) and mixed venous saturation (SvO_2_) in endotoxemic groups had different trajectories during the experiment compared to non-endotoxemic controls in the NaCl + NaCl group (*p* < 0.01 vs. *p* < 0.05 respectively). Left ventricular stroke work index (LWSVI) was lower in each endotoxemic group compared to the NaCl + NaCl group (*p* < 0.01) and was also lower in the Etx + NaCl group vs. the Etx + Hct and the Hct + Etx groups (*p* < 0.01 for both). Plasma NGAL correlated to HR (rho = 0.53), MAP (rho = −0.37), MPAP (rho = 0.75), CI (rho = −0.39), SvO_2_ (rho = −0.54), and LVSWI (rho = −0.60) at 3 h when circulatory derangement was most pronounced. At the end of the experiment, plasma NGAL correlated to HR (rho = 0.55), MAP (rho = 0.01), MPAP (rho = 0.55), CI (rho = −0.33), SvO_2_ (rho = −0.30), and LVSWI (rho = −0.45).

**Table 2:**
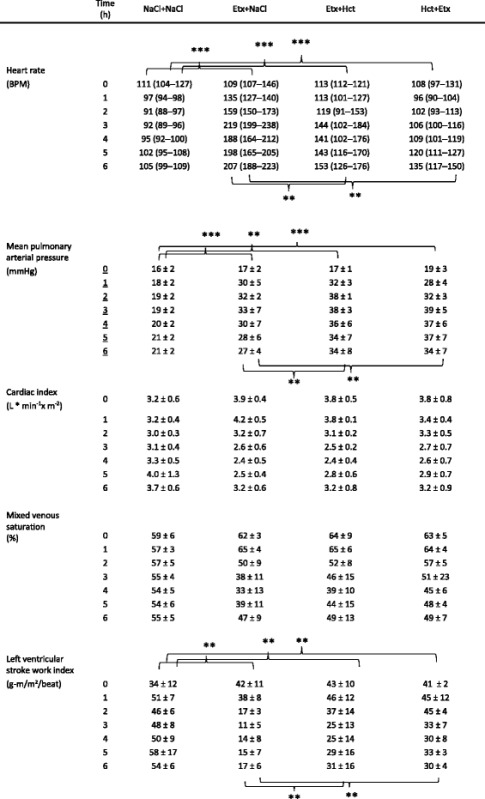
Circulatory variables during 6 h of endotoxemia. Mean ± SD or median (IQR)

**p < 0.01; ***p < 0.001 denote intergroup differences 1–6 h

### Lactate and base excess

Plasma lactate was lower in non-endotoxemic controls (the NaCl + NaCl group) compared to endotoxemic groups (*p* < 0.001) with no other intergroup differences (Table 3). Base excess was higher in the NaCl + NaCl vs. each endotoxemic group (*p* < 0.001), and was also higher in the Hct + Etx vs. the Etx + NaCl groups (*p* < 0.01) and the Etx + Hct group (*p* < 0.01).

**Table 3:**
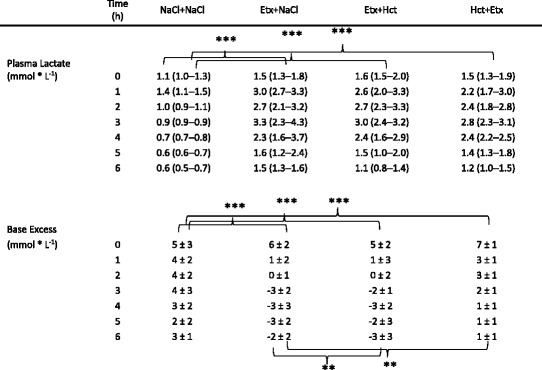
Plasma lactate and base excess during 6 h of endotoxemia. Mean ± SD or median (IQR).

**p < 0.01; ***p < 0.001 denote inter group differences 1–6 h

## Discussion

### Key findings

Plasma NGAL increases during systemic inflammatory response triggered by endotoxin infusion. This inflammatory response is attenuated for several hours by hydrocortisone administration prior to the start of endotoxin infusion suggesting that the effect on plasma NGAL levels is a very early phenomenon in endotoxemia. Hydrocortisone treatment did not affect urine NGAL levels in endotoxemic pigs. NGAL levels correlated (inversely) to neutrophil granulocyte count but not urine output, suggesting that the NGAL response to endotoxemia was primarily related to the extent of systemic inflammation, not to kidney injury. Endotoxemic animals treated with hydrocortisone had higher mean arterial pressure, lower heart rate, lower mean pulmonary arterial pressure, and higher left ventricular stroke work index than those without such treatment, and circulatory derangement was associated with high plasma NGAL levels.

### Previous studies

The anti-inflammatory effect of glucocorticoids in septic systemic inflammation is well documented, and in a previous experimental study of ours [18], we concluded that steroid treatment prior to endotoxemia had beneficial circulatory effects, which were not mediated by TNF-alpha, IL-6, or NO. Although it is a matter of debate, hydrocortisone is advocated to treat septic shock [19, 20]. Timing of steroid administration could have impact on the survival rate [21]. In this study, we hypothesized that one of the anti-inflammatory mechanisms involved in endotoxin-induced systemic inflammatory response is the attenuation of neutrophil granulocyte activation. NGAL is concentrated within the granules of neutrophil granulocytes. These granules are released into the plasma upon substantial inflammatory stimulation [22] where NGAL exerts bacteriostatic effects [6] and activates neutrophil granulocytes among other functions. Although several other cell types are potential sources of NGAL [23], our data suggest that plasma NGAL is strongly associated to the neutrophil granulocytes. No association between NGAL and renal function was seen. Others have also reported NGAL to be a marker of inflammatory response rather than to that of sepsis [24–26] and specifically pointed out that the origin of NGAL in systemic inflammatory response is neutrophil granulocytes [27]. Endotoxin induces this release of NGAL from these cells via the Toll-like receptor 4 (TLR-4) [28].

To our knowledge, the diminished increase in plasma NGAL with pretreatment with hydrocortisone in the current study has not been reported previously in endotoxemia or sepsis. However, these findings correspond to those in other conditions such as AKI, where reduced oxidative stress [29] and attenuated neutrophil granulocyte response were suggested as possible mechanisms of steroid pretreatment on NGAL levels [30]. Steroids could potentially inhibit the production of NGAL though the IKKB/Nf-KB pathway, which is suppressed by these drugs [31]. We have previously described that hydrocortisone, given to endotoxin-exposed pigs [18], leads to increased circulatory stability in endotoxemic shock and chose NGAL to investigate further the role of neutrophil granulocyte activation. The current experiments show attenuated circulatory and acid-base response to endotoxin in pigs treated with hydrocortisone, but this effect was only partly related to the timing of this treatment. In contrast, the inflammatory response including lower NGAL levels and higher white blood cell counts if hydrocortisone was administered prior to the endotoxin infusion, but not later. This may be contradictory since the release of NGAL occurs early in granulocyte differentiation [7], and given the high turnover and the short half-life of these cells in endotoxemia [32] as well as the short half-life of NGAL [33], one would have expected that the timing of hydrocortisone should not have impacted on neutrophil activation in these experiments. However, released NGAL has not only bacteriostatic functions, but also has an important role in the maturation and activation of neutrophil granulocytes [34, 35], i.e., NGAL-mediated neutrophil activation is a positive feedback loop triggered by e.g., endotoxemia. We therefore postulate that decreased NGAL levels may inhibit the additional recruitment of neutrophils i.e., limiting the inflammatory response. The importance of NGAL in the neutrophil-mediated inflammatory response has been described in NGAL-deficient mice and men [35].

Finally, we did not find a consistent effect of timing on the circulatory effects of hydrocortisone in our study. A possible explanation could be that these effects, apart from the inflammatory response may also be mediated by other mechanisms, e.g., inducible NO synthase [36]. However, there was a strong association between plasma NGAL and circulatory failure induced by endotoxin in this experiment.

### Strengths and limitations

This is, as far as we know, the first report on the effect and timing of hydrocortisone administration on NGAL levels in plasma, linking it to neutrophil granulocyte counts in endotoxemia. Other strengths include that our findings on the effect of timing of hydrocortisone replicate previous findings. The results are further supported by the design with four groups including control groups for the effects of endotoxin and of hydrocortisone.

However, the study has a number of limitations. Firstly, the aim of the study was to study the effects of steroids in sepsis; the study was conducted in an endotoxemic model on young and previously healthy animals limiting its validity for patients seen in intensive care. Nevertheless, endotoxemia induces the innate immune system, as does sepsis, and investigating the timing of steroid administration is difficult to do in the clinical situation. Also, these young and healthy animals may have different physiological response to the stress dose of hydrocortisone used in our protocol compared to patients in intensive care that are generally older and are more prone to have comorbidities. Secondly, although there was a total of 16 animals included in the study, there were only four animals in each group. Although this limits the power of the study, it has been taken into account, in the discussion of positive results, minimizing the effect of beta error. In addition, an obvious limitation of the study is the short observation period. Timing of hydrocortisone administration may be of limited impact on the systemic inflammatory response over a period of days, which is the natural course of sepsis. Finally, the correlation between neutrophil granulocytes and plasma NGAL is merely an association, and NGAL may have other sources; however, our findings are in line with the current literature suggesting neutrophil granulocytes to be the main source of NGAL in sepsis.

### Clinical implications

Our study could indicate the value of pre-emptive and possibly early, low-dose steroid treatment to limit systemic inflammatory response. Another clinical implication of this study is to point out the possibility that patients on chronic steroid treatment initiated before the onset of a severe infection leading to a septic condition, may exhibit less obvious clinical signs of sepsis, thus obscuring this diagnosis. Although this study addresses NGAL as an inflammatory response mediator, this molecule has important bacteriostatic properties. Hydrocortisone administration attenuates the effects of endotoxemia on the circulation, but low NGAL levels may also lead to diminished microbial defense, a phenomenon described in clinical studies in septic shock patients [37]. Monitoring plasma NGAL could be a method to follow steroid-induced immunosuppression.

### Future studies

Studies with longer observation periods with repeated steroid doses as well as studies in humans would increase understanding of how timing of steroid treatment influences the anti-inflammatory effects of steroids.

## Conclusions

Administration of steroids, prior to endotoxemia, induces reduced plasma concentration of NGAL which may be one mechanism by which corticosteroids exert their anti-inflammatory activity and attenuate endotoxin-induced circulatory failure. The NGAL response may be linked to the inflammatory response of neutrophil granulocytes.
